# The Analysis of Cu(II)/Zn(II) Cyclopeptide System as Potential Cu,ZnSOD Mimic Center

**DOI:** 10.1007/s10989-017-9574-8

**Published:** 2017-02-16

**Authors:** Aleksandra Kotynia, Tomasz Janek, Żaneta Czyżnikowska, Sylwia Bielińska, Wojciech Kamysz, Justyna Brasuń

**Affiliations:** 1 Department of Inorganic Chemistry, Wroclaw Medical University, Borowska 211a, 50-552 Wroclaw, Poland; 2 Department of Inorganic Chemistry, Medical University of Gdansk, 80-416 Gdansk, Poland

**Keywords:** Heteronuclear copper(II) and zinc(II) complexes, Potentiometry, Stability constants, SOD activity, Cu ZnSOD mimetics

## Abstract

In this paper are presented the features of copper (II) and zinc (II) heteronuclear complexes of the cyclic peptide—c(HKHGPG)_2_. The coordination properties of ligand were studied by potentiometric, UV–Vis and CD spectroscopic methods. These experiments were carried out in aqueous solutions at 298 K depending on pH. It turned out that in a physiological pH dominates Cu(II)/Zn(II) complex ([CuZnL]^4+^) which could mimic the active center of superoxide dismutase (Cu,ZnSOD). In next step we performed *in vitro* research on Cu,ZnSOD activity for [CuZnL]^4+^ complex existing in 7.4 pH by the method of reduction of nitroblue tetrazolium (NBT). Also mono- and di-nuclear copper (II) complexes of this ligand were examined. The ability of inhibition free radical reaction were compared for all complexes. The results of these studies show that Cu(II) mono-, di-nuclear and Cu(II)/Zn(II) complexes becoming to new promising synthetic superoxide dismutase mimetics, and should be considered for further biological assays.

## Introduction

In recent years there has been observed a rapid expansion in the development of Cu(II) complexes as important bioactive compounds as potential drugs for therapeutic intervention in various diseases. Their properties as radiopharmaceuticals (Iakovidis et al. 2011), antimicrobial (Jimenez-Garrido et al. 2005), anti-inflammatory (Govind and Rajesh 1992), antitumor (Travnicek et al. 2001) agents, enzyme inhibitors (Lachowicz et al. 2015) or chemical nucleases (Efthimiadou et al. 2007) have been reported for numerous Cu(II) complexes.

A wide role of transition metal ions in biological processes induces the intensive development of studies allowing to the characteristic of copper homeostasis. These studies are performed by the using of the biomimetics mimicking the structure or activity of the naturally occurring systems. Copper plays significant role in the biological systems where is mainly bound to the peptides or proteins. That interaction with peptide/protein is the base of their transport in living systems and storage. Moreover, this interaction is responsible for the activity of number of metalloperoteins or their toxicity.

The binding of copper metal ions by peptide may be realized by coordination to donors from the peptide chain: nitrogen of the N-terminal amino group or peptide bond and oxygen form the C-terminal carboxylate group or peptide bond. However, in the living systems, it takes place mainly to donor atoms of side chains of the amino acid residues e.g. His or Asp (Kozlowski et al. 2005; Sigel and Martin 1982; Sovago et al. 2012). The involvement of donors from the side chains group of amino acid residues is found inter alia in the active centers of metalloenzymes with one (e.g. plastocyanin, nitrite reductase, quercetinase) or two metal ions (e.g. tyrosinase, hemocyanin, cytochrome c oxidase) (MacPherson and Murphy 2007; McCleverty and Meyer 2004).

**Fig. 1:**
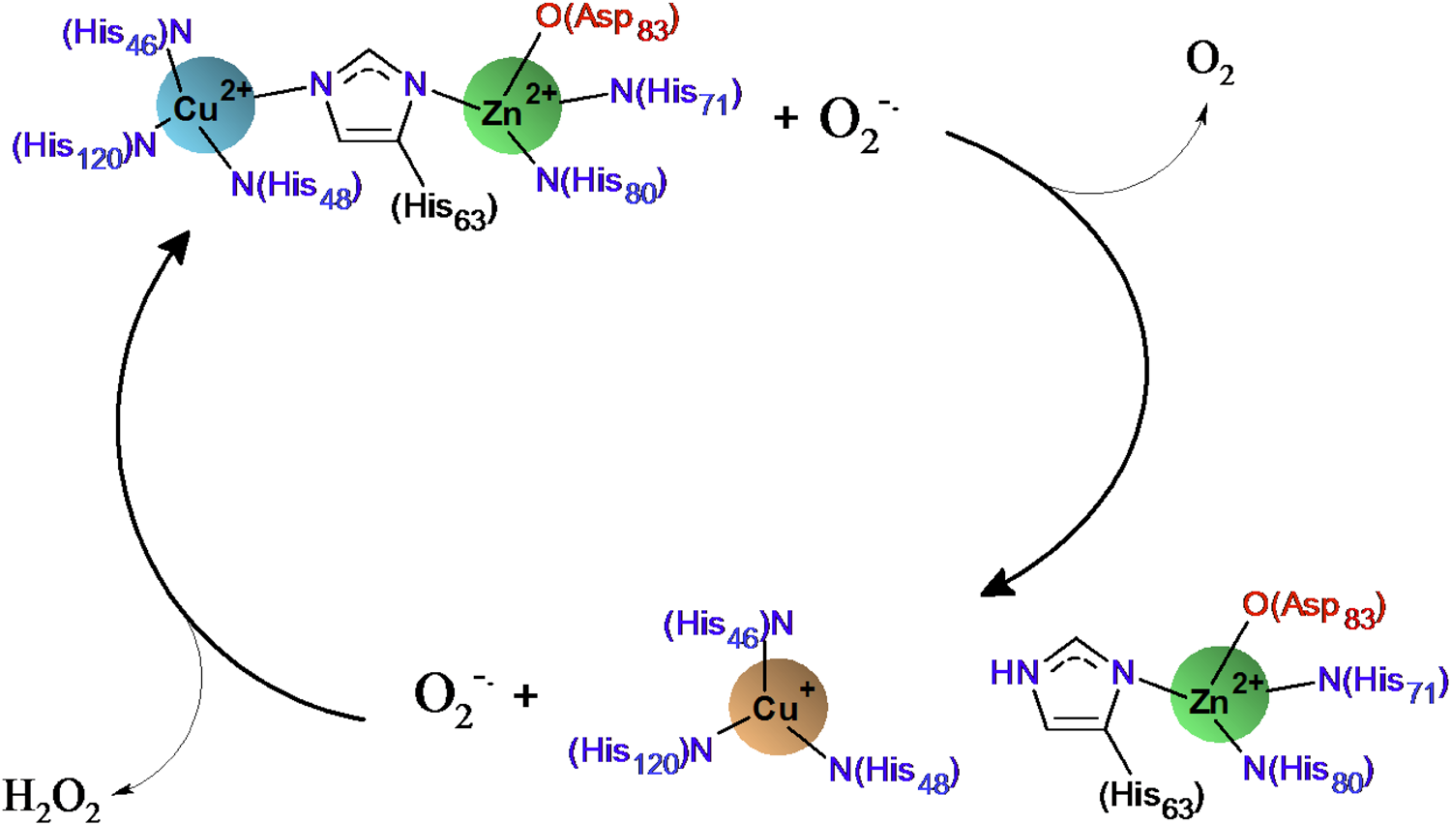
The schematic mechanism of the metallo-active center of Cu,ZnSOD (Hart et al. 1999; MacPherson and Murphy 2007)

Several authors have brought the attention to the redox properties (Fragoso et al. 2015) as well as antioxidant/superoxide scavenging of Cu(II) complexes (Fragoso et al. 2015; Pap et al. 2011). The copper (II) complexes exhibit antioxidant properties due to their capacity to mimic the activity of the superoxide dismutase enzyme. The mononuclear and dinuclear copper (II) complexes with multihistidine peptides are good functional models of metallo-enzymes center *e,g*, the Cu,ZnSOD (Bonomo et al. 1998; Timari et al. 2011; Valensin et al. 2009) and phenoloxidase enzyme (Matusiak et al. 2014). Superoxide dismutase, Cu,ZnSOD is a metalloprotein where the copper is present in the catalytic center and undergoes reduction–oxidation cycling during the dismutation of superoxide anion. Although zinc ion is not involved in redox cycle, it facilitates the oxidation step by maintaining the configuration of the active site (Agotegaray et al. 2012). The schematic mechanism of superoxide dismutation is present on Fig. 1.

The cyclopeptides are the subtype of the peptides having characteristic cyclic motif in their structure which may be obtained by formation of the disulphide bond between two Cys residues or formation of the peptide bond. The cyclization of the peptide chain influences significantly the abilities of the peptides (Bockus et al. 2013; Hill et al. 2014) e.g. the peptides with the cyclic structure are more stable in the biological conditions than the linear analogues (Kumar et al. 2006; Roxin and Zheng 2012). Moreover the cyclic structure of the peptide chain makes it less flexible and influences the steric arrangement of the side chains groups (Hruby 1982; Hruby et al. 1990) as well as coordination abilities (Brasun et al. 2004; Fragoso et al. 2013). The copper (II) binding by cyclopeptides strongly depends on the acid–base properties of the side chain groups (Czapor et al. 2011; Matera et al. 2008), its optical isomerism (Matera et al. 2008), number of His residues in the peptide chain as well as the size of the peptide ring (Brasuń et al. 2009). Moreover, the insertion of two Pro amino acid residues in their sequence allows for creating of two well defined binding sites for metal ions (Kotynia et al. 2012, 2014). Our previous studies have shown that the presented cyclopeptide (Kotynia et al. 2014) as well as its analogue with the c(HKHPHKHP) sequence (Kotynia et al. 2012) are able to form di-copper complexes with the {2N_Im_, 2$\text{N}_{\text{am}}^{-}$} binding manner observed in the physiological range of pH. Based on previous studies we decided to characterize the abilities of cyclopeptide with the c(HKHGPG)_2_ sequence to form the heteronuclear, Cu(II)/Zn(II) complexes as well as the Cu,ZnSOD-like activity.

## Experimental Methods

### The Peptide Synthesis

The synthesis of discussed peptide c(HKHGPG)_2_ have been described in details in the previously published article (Kotynia et al. 2014).

### The Potentiometric Measurements

The potentiometric titration were performed on Molspin pH-meter system using a Mettler Toledo InLab®Micro combined electrode. The electrode was calibrated by titration hydrogen ion sample using HCl three times before each system. The KOH titrant (0.1 mol dm^− 3^) was added from a 0.250 ml micrometer syringe and the concentration was calibrated by weight titrations of standard materials. All samples were titrated in thermostat vessel at 298 K over the pH range 2.5–11.5 under argon gas atmosphere. The ligand concentration was 8 × 10^− 4^ mol dm^− 3^ in solution the ionic strength was in 0.1 mol dm^− 3^ KCl. The samples with metals ion system were prepared by added equimolar amounts of CuCl_2_ and ZnCl_2_ solutions where final ratio Cu(II):Zn(II):L = 1:1:1. The stability constants of the proton and metal complexes were calculated from titration curves by the HYPERQUAD 2008 (written by Peter Gans, Protonic Software) computer program (Gans et al. 1996). The standard deviations for each constants were computed and refer to random errors only. They were a good indication of the importance of a particular species in the equilibrium.

### The UV–Vis and CD Spectroscopic Measurements

The absorption spectra of complexes were recorded on Varian Carry 50 Bio spectrophotometer in 1 cm path length quartz cells. All UV–Vis spectra were collected in the 200–900 nm range. The spectroscopic measurements were carried out at 298 K, samples solutions were similar concentrations as in pH-metric titration. The circular dichroism (CD) spectra were recorded on a magnetic circular dichroism JASCO J-1500 spectrometer in 230–800 nm range, using 1 cm cuvettes.

### The Measurement of Cu,ZnSOD Activity

The in vitro SOD activity of Cu(II)/Zn(II) cyclopeptide complexes was determined by the method of Beauchamp et al. (Beauchamp and Fridovich 1971). The tests were carried out at 25 °C, in samples containing Cu(II) and Zn(II) peptide complexes in different molar ratios in Tris–HCl buffer (25 mM, pH 7.4). The enzymatic assay contained (5 × 10^− 5^ mol dm^−3^) nitroblue tetrazolium (NBT), (10^− 3^ mol dm^−3^) xanthine, and an appropriate amount of xanthine oxidase in order to cause a change in absorbance at 560 nm of about Δ*A*_560_ = 0.020 min^− 1^. The NBT reduction rate was measured in the presence and the absence of the investigated system for 300 s. The SOD-like activity was then expressed by the IC_50_ values (the concentration that causes 50% inhibition of NBT reduction).

### Molecular Modelling

In order to propose the possible structure of investigated complexes we have examined tens of different conformers of [ZnCuL]^4+^, [ZnCuH-_1_L]^3+^, [ZnCuH-_2_L]^2+^, [ZnCuH-_3_L]^+^ species. Geometries of all complexes have been fully optimized using NDDO-type semiempirical method referred to as PM6 which offers reasonable compromise between accuracy and computational cost (Stewart 2009). As it has been shown by Stewart, in the case of many properties (including equilibrium geometries, electric-dipol properties and heats of formation) PM6 method improves substantially upon PM3 method. This method is also quite useful in determining structures of bioinorganic complexes with transition metals and modeling of proteins and metaloproteins (Stewart 2009). Geometry optimizations have been followed by evaluation of hessian matrix to confirm that obtained structures correspond to minima on the potential energy surface. To account for solvent effects (water solution) we have applied polarizable continuum model (PCM) (Cances et al. 1997; Tomasi et al. 2005, 1999). The lowest energy conformers obtained using the PM6 method were presented in the Fig. 4. All calculations have been performed within unrestricted formalism using Gaussian 09 suite of programs (Frisch et al. 2009).

## Results

### The Formation of Heteronuclear Cu(II)/Zn(II) Complexes

The analyzed cyclopeptide has two -GHKHG- binding sites potential for metal ions. The previous studies performed for the system with investigated peptide and double excess of Cu(II) ions have shown that it is able to form di-copper complexes. This process is observed above pH 6. Below this pH only mononuclear species, with imidazole donors involved in metal binding, exist in the system. Furthermore, in the physiological range of pH the dominant species is the complex with the {2N_Im_, 2$\text{N}_{\text{am}}^{-}$}/{2N_Im_, 2$\text{N}_{\text{am}}^{-}$} coordination mode for both copper (II) ions and it does not change with increasing of pH (Kotynia et al. 2014).

**Fig. 2:**
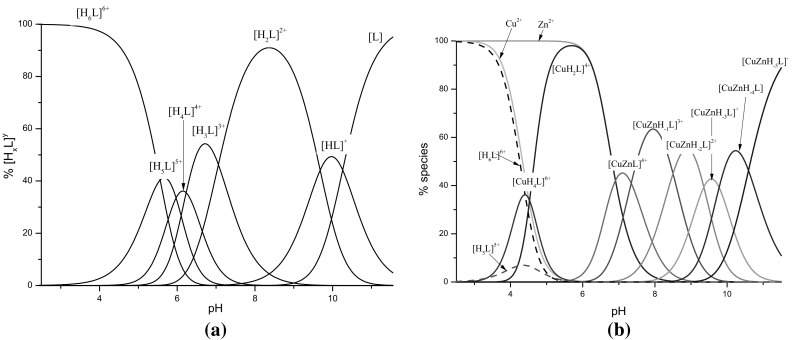
The distribution of species: **a** for free ligand c(HKHGPG)_2_. **b** Heteronuclear complexes formed in Cu(II)/c(HKHGPG)_2_/Zn(II) = 1:1:1 system, depending on the pH

In the present manuscript we show the results of potentiometric and spectroscopic studies for the Cu(II)/c(HKHGPG)_2_/Zn(II) system. The potentiometric results are presented in Table 1 and Fig. 2 whilst the spectroscopic data in Table 2. The investigated ligand is the H_6_L acid with four His and two Lys amino acid residues protonated (Table 1; Fig. 2a). With increase of pH [H_5_L]^5+^, [H_4_L]^4+^, [H_3_L]^3+^, [H_2_L]^2+^ forms of the ligand appear in the system what can be assigned to the protons dissociation from the imidazoles of His residue. The formation of two last forms [HL]^+^and [L] results by proton dissociation from side chains of both Lys residues.

**Table 1:** The stability constants for c(HKHGPG)_2_ obtain from HYPERQUAD calculation and its Cu(II)/Zn(II) heteronuclear species with comparison with copper (II) mono- and di-nuclear complexes

log*β*	HL	H_2_L	H_3_L	H_4_L	H_5_L	H_6_L
	10.26 ± 0.012	19.94 ± 0.009	27.00 ± 0.012	33.23 ± 0.015	39.20 ± 0.013	44.76 ± 0.014

log*K*	NH_2_ (Lys)	NH_2_ (Lys)	N_Im_ (His)	N_Im_ (His)	N_Im_ (His)	N_Im_ (His)
	10.26	9.68	7.06	6.23	5.97	5.12

Cu (II) system (Kotynia et al. 2014)		Cu(II)/Cu(II) system (Kotynia et al. 2014)		Cu(II /Zn(II) system		
Species	log*β*	Species	log*β*	Species	log*β*	
CuH_4_L	39.20	CuH_4_	39.20	CuH_4_L	38.88 ± 0.016	
CuH_2_L	29.99	CuH_2_L	29.99	CuH_2_L	29.82 ± 0.010	
CuL	14.77	Cu_2_H-_1_L	14.54	ZnCuL	19.38 ± 0.018	
CuH-_1_L	5.54	Cu_2_H-_2_L	8.26	ZnCuH-_1_L	12.04 ± 0.015	
CuH-_2_L	−4.57	Cu_2_H-_3_L	−1.54	ZnCuH-_2_L	3.54 ± 0.020	
CuH-_3_L	−14.69	Cu_2_H-_4_L	−11.64	ZnCuH-_3_L	−5.82 ± 0.022	
				ZnCuH-_4_L	−15.60 ± 0.021	
				ZnCuH-_5_L	−26.19 ± 0.021	

	pK		pK		pK	
log*β*_CuH4L_ − log*β*_CuH2L _	9.21	log*β*_CuH4L _ − log*β*_CuH2L _	9.21	log*β*_CuH4L _ − log*β*_CuH2L _	9.06	
log*β*_CuH2L_ − log*β*_CuL _	15.25	log*β*_Cu2H-1L _ − log*β*_Cu2H-2L _	6.28	log*β*_ZnCuL _ − log*β*_ZnCuH-1L _	7.34	
log*β*_CuL _− log*β*_CuH-1L _	9.23	log*β*_Cu2H-2L _ − log*β*_Cu2H-3L _	9.80	log*β*_ZnCuH-1L _ − log*β*_ZnCuH-2L _	8.50	
log*β*_CuH-1L_− log*β*_CuH-2L_	10.11	log*β*_Cu2H-3L _ − log*β*_Cu2H-4L _	10.10	log*β*_ZnCuH-2L _ − log*β*_ZnCuH-3L _	9.36	
log*β*_CuH-2L_ − log*β*_CuH-3L_	10.12			log*β*_ZnCuH-3L _ − log*β*_ZnCuH-4L _	9.78	
				log*β*_ZnCuH-4L _− log*β*_ZnCuH-5L _	10.59	

The charges of species have been omitted to more clarity

**Table 2:** The UV–Vis and CD spectroscopic properties for complexes formed in Cu(II)/c(HKHGPG)_2_/Zn(II) = 1:1:1 system

pH	Prevailing Cu(II) coordination mode		UV–Vis		CD	
The percentage of species existing in each pH			λ [nm]	ε[dm^3^ cm mol^−1^]	λ [nm]	∆ε [dm^3^ cm mol^− 1^]
5.5	{4N_Im_}		585	58	304.9^c^	−0.08
98%—CuH_2_L, 2%—H_5_L					269.4^c^	+0.14
7.0	{2N_Im_, 2$\text{N}_{\text{am}}^{-}$}		547^a^	102	560.1^a^	−0.11
			603 sh		487.5^a^	+0.14
43%—CuZnL,					343.3^b^	−0.60
34%—CuH_2_L, 23%—CuZnH-_1_L					280.4 sh	
					252.0^c^	+1.67
8.0	{2N_Im_, 2$\text{N}_{\text{am}}^{-}$}		537^a^	114	552.7^a^	−0.17
			609 sh		481.0^a^	+0.15
63%—CuZnH-_1_L					343.8^b^	−0.83
2%—CuH_2_L, 14%—CuZnL					280.4 sh	
20%—CuZnH-_2_L, 1%—CuZnH-_3_L					244.1^c^	+3.16
~11.0	{N_Im_, 3$\text{N}_{\text{am}}^{-}$}		530^a^	120	661.6^a^	+0.25
			618 sh		518.5^a^	−0.31
72%—CuZnH-_5_L					343.3^b^	−0.21
27%—CuZnH-_4_L, 1%—CuZnH-_3_L					261.4^c^	+1.86

The charges of species have been omitted to more clarity

^a^d – d transition, *sh* shoulder

^b^ *N* ^*−*^ *→ Cu* ^*2+*^ charge transfer

^c^ *N*_*Im*_ *→ Cu* ^*2+*^ charge transfer

**Fig. 3:**
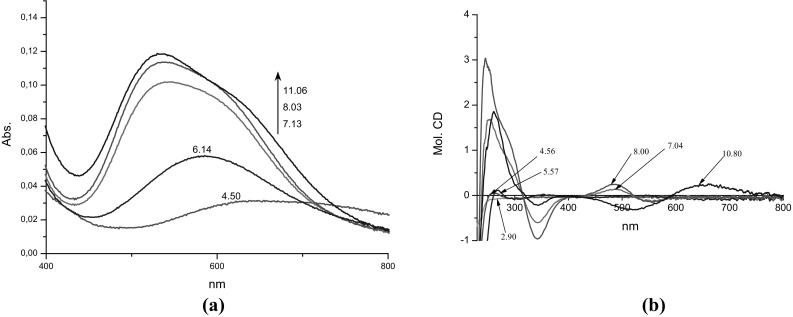
The **a** UV–Vis. **b** CD spectra of complexes in Cu(II)/c(HKHGPG)_2_/Zn(II) = 1:1:1 system, depending on the pH

The metal binding starts around pH 4 by complexation only copper (II) ions and formation two mononuclear complexes: [CuH_4_L]^6+^ and [CuH_2_L]^4+^ (Fig. 2b). The stabilities constants calculated for both complexes (Table 1) are comparable to the constants of corresponding complexes with the {2N_Im_} and {4N_Im_} binding modes, consecutively, which are found in the system with the Cu(II)-to-ligand molar ratio 1:1 (Table 1) (Kotynia et al. 2014). Due to the low concentration of the [CuH_4_L]^6+^ species, it was possible to obtain the spectroscopic parameters only for the [CuH_2_L]^4+^ complex. The analysis of the potentiometric results for the metal ions—system shows, that at pH 2.9 exists only free ligand (Fig. 2b) and the CD spectrum recorded at this pH significantly differs from the spectrum obtained at pH 5.57. It strongly supports that the CD spectrum (at pH 5.57) can be attributed to the [CuH_2_L]^4+^ complex. The presence of two CT bands: one negative at 304 nm and second positive at 269 nm in the CD spectrum confirms the involvement only imidazole donors in copper (II) binding (Table 2; Fig. 3b).

With increase of pH, two protons dissociate and the first, heteronuclear [CuZnL]^4+^ complex is formed. Its appearance significantly influences the spectral abilities of the system, primarily the CD spectrum. Owing to the fact, that the Zn(II) cation is the d^10^-metal ion and it is spectrophotometrically silent, the changes in the spectral abilities of the solution correspond to changes in the coordination sphere of Cu(II) ion. The appearance of the negative CT band at 343 nm in CD spectrum obtained at pH 7 (Table 2; Fig. 3b) supports the involvement of amide nitrogens in copper (II) binding and the location of the main d–d band at 547 nm in UV–Vis spectrum shows binding of four nitrogens to the copper (II). Based on these facts the {2N_Im_, 2$\text{N}_{\text{am}}^{-}$} may be proposed for Cu(II) ion. On the other hand, there are two possibilities of Zn(II) ion binding in discussed complex: by one or two imidazole nitrogens. The value of corrected log*β* ^*^ = 4.61, were log*β* ^*^ = log*β*_ZnCuL_–log*β*_CuL_, is comparable for the stability constants of the Zn(II) complexes with two imidazole nitrogens bound to metal ion (Kallay et al. 2009; Valensin et al. 2009). The presented assumptions allow to propose the Cu(II) {2N_Im_, 2$\text{N}_{\text{am}}^{-}$}/Zn(II){2N_Im_, 2H_2_O} binding modes of both metal ions in the [CuZnL]^4+^ species.

The formation of next two complexes, [CuZnH_-1_L]^3+^ and [CuZnH_-2_L]^2+^ does not significantly influence the spectral abilities of the system (Fig. 3; Table 2). It supports the same coordination sphere of copper (II) ion. The formation of these species may be addressed to proton dissociation from water molecules bound to Zn(II) ion and formation the hydroxo complexes: [CuZnL(OH^−^)(H_2_O)]^3+^ and [CuZnL(OH^−^)_2_]^2+^. The value of log*K*_*CuZnL → CuZnH*-*1L*_ = 7.34 for proton dissociation from first H_2_O molecule is relatively low but it is probably connected to the hydrolityc activity of Zn(II) complexes (Jakab et al. 2008; Kallay et al. 2009; Valensin et al. 2009).

With the increase of the pH, the next proton dissociates from the [CuZnH_-2_L]^2+^ complex and the [CuZnH_-3_L]^+^ species appears in the system and the spectral abilities of the system are changing (Table 2; Fig. 3). The CD parameters are comparable to those obtained for the copper complexes with one imidazole and three amide donors in the plane: {N_Im_, 3$\text{N}_{\text{am}}^{-}$} found in the system with the equimolar conditions (Kotynia et al. 2014). Due to this fact, the Cu(II) {N_Im_, 3$\text{N}_{\text{am}}^{-}$}/Zn(II){2N_Im_, 2OH^−^}binding mode can be proposed for discussed species.

As the last, two complexes, [CuZnH_-4_L] and [CuZnH_-5_L]^−^exists in the system. Their formation is connected to the proton dissociation from side chain amino groups of both Lys amino acid residues what is supported by the values of the log*K*_*CuZn*-*3L→CuZnH*-*4L*_ = 9.78 and log*K*_*CuZn*-*4L→CuZnH*-*5L*_ = 10.59 (Table 1), however the coordination sphere of both metals does not change.

**Fig. 4:**
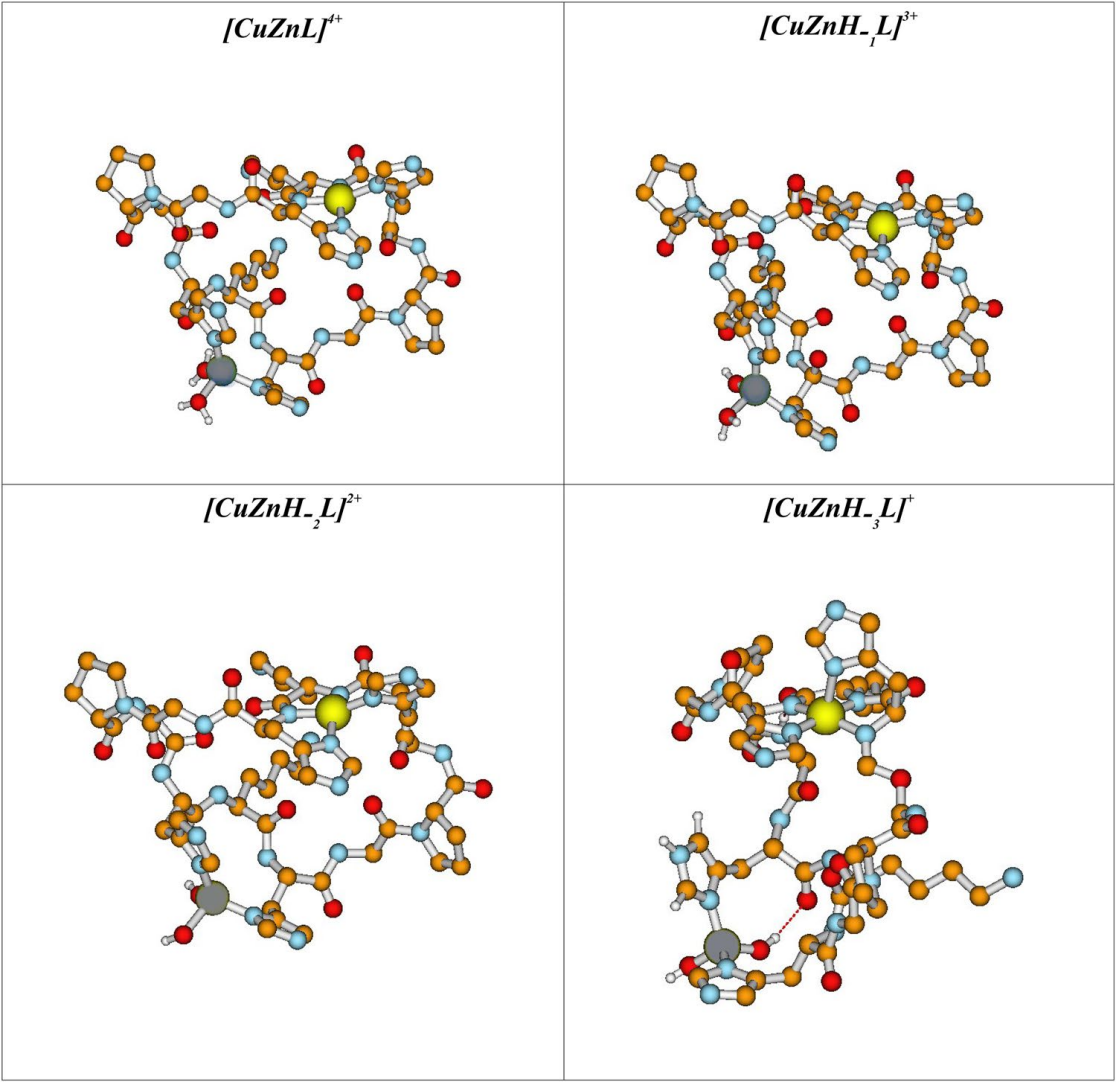
Lowest-energy conformer of [CuZnL]^4+^, [CuZnH-_1_L]^3+^, [CuZnH-_2_L]^2+^, [CuZnH-_3_L]^+^ form of c(HKHGPG)_2_ peptide obtained using theoretical calculations. All hydrogens atoms are removed for the sake of clarity. Copper is shown in yellow, zinc in grey, nitrogens in cyan, oxygens in red and carbon atoms in orange

In order to analyze the structural aspects of binding properties of c(HKHGPG)_2_ towards copper (II) and zinc (II) ions the molecular modeling studies were performed. The possible conformations of Cu(II)/c(HKHGPG)_2_/Zn(II) complexes at different pH were optimized at PM6 level of theory leading to many unique conformations. In this paragraph, we report only on the most stable conformers of Cu(II)/c(HKHGPG)_2_/Zn(II)-peptide complexes in different ligands protonation states. Figure 4 contains the lowest-energy structures. The obtained structures are characterized by structural parameters which are in agreement with data obtained from X-ray diffraction measurements collected in CSD database (Allen et al. 1983).

The experimental results presented above have shown, that the creation of first heteronuclear, [CuZnL]^4+^, complex is connected with dissociation of two, copper-bound, imidazole donors. Next, the deprotonation and involvement of two amide nitrogens in Cu(II) binding lead to the formation of the species with the {2N_Im_, 2$\text{N}_{\text{am}}^{-}$} binding mode. Moreover in this complex Zn(II) ion is coordinated by two imidazole nitrogens and two water molecules {2N_Im_, 2H_2_O} in tetrahedral configuration. Moreover, the formation of the next two, [CuZnH_−1_L]^3+^ and [CuZnH_−2_L]^2+^ species results from the deprotonation of water molecules bound to Zn(II) ion. This process does not influence the structural properties of Cu(II) binding mode. The distances Cu–N_Im_ and Cu–N_amide_ in considered cases are almost the same and dihedral angles between donor nitrogens ($\text{N}_{\text{am}}^{-}$ $\text{N}_{\text{am}}^{-}$ $\text{N}_{\text{im}}^{-}$ $\text{N}_{\text{im}}^{-}$) amount approximately 25°.

According to the potentiometric and spectroscopic results, the last calculated structure was the [CuZnH_− 3_L]^+^structure, in which the Cu(II) ion is coordinated by four nitrogen donors: one N_Im_ and three amides in the plane as it was found in the calculated structure of the [CuH_− 3_L]^+^ in copper (II) mononuclear system (Kotynia et al. 2014).

Nevertheless, in the case of the Cu(II)/Zn(II) complex copper is coordinated by two imidazole and three amide in almost tetragonal pyramid configuration.

### The Cu,ZnSOD Activity Measurements

**Fig. 5:**
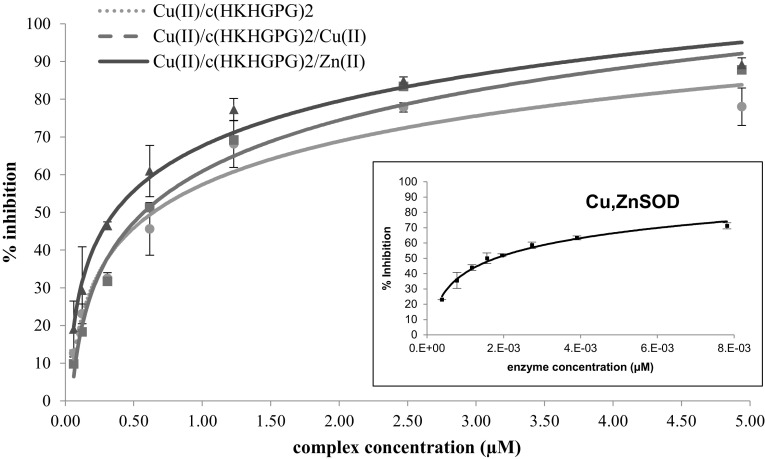
Inhibition of the nitroblue tetrazolium (NBT) reduction by superoxide as a function of the concentration of His_4_-cyclopeptides complex with Cu(II) and Zn(II), ±SD n = 15

The main goal of the presented studies was the investigation of the cyclopeptide as the potential tool for creation of the mimetics of dinuclear active centers of metalloproteins. We have performed the preliminary studies on the Cu,ZnSOD activity of three systems with discussed cyclopeptide: Cu(II)/c(HKHGPG)_2_/Zn(II) with the Cu(II) {2N_Im_, 2N^−^}, {4N_Im_} binding modes, Cu(II)/c(HKHGPG)_2_ with {4N_Im_} and Cu(II)/c(HKHGPG)_2_/Cu(II) with {2N_Im_, 2 N^−^}, {2N_Im_, 2N^−^}(Kotynia et al. 2014) system at pH 7.4. In order to prove that the studied Cu(II)/Zn(II) cyclopeptide complexes can decompose the superoxide anion, the Cu,ZnSOD activities were investigated by the NBT assay in Tris–HCl buffer at physiological pH. The superoxide anion was generated *in situ* by the xanthine/xanthine oxidase reaction and spectrophotometrically detected by monitoring the reduction of NBT. The concentrations of the Cu(II) and Cu(II)/Zn(II) complexes required to attain 50% inhibition of the reduction (defined as IC_50_) were determined. The IC_50_ values characterizing the Cu,ZnSOD activity of the complexes were calculated based on saturation curves (Fig. 5). The greatest activity was observed for the native Cu,ZnSOD enzyme, which was taken as the reference point for the other measurements. The most efficient complex was Cu(II)/(HKHGPG)_2_/Zn(II) complexes ([CuZnL]^4+^). Nonetheless, all peptide complexes were found to be redox-active, demonstrating their effective ability to scavenge the superoxide anion. The IC_50_ values as well as their relative activity (%) compared to the native Cu,ZnSOD are shown in Table 3. The heteronuclear complexes which are dominated in physiological pH have smaller IC_50_ value then their copper (II) mono- and di-nuclear complexes. What it probably cases by binding of Zn(II) ion.

**Table 3:** IC_50_ (μΜ) values of the Cu,ZnSOD-mimicking Cu(II)/c(HKHGPG)_2_/Zn(II) complexes at pH 7.4

Complex	IC_50_ (µM)	% Relative activity	Species	Prevailing Cu(II) coordination mode
Cu(II)/(HKHGPG)_2_; 1:1	0.643	0.28	CuH_2_L, CuL, Cu_2_H_-2_L	{4N_Im_}, {2N_Im_, 2N^−^}
Cu(II)/(HKHGPG)_2_; 2:1	0.574	0.31	Cu_2_H_-2_L	{2N_Im_, 2N^−^}
Cu(II)/(HKHGPG)_2_/Zn(II); 1:1:1	0.360	0.50	CuZnL	{2N_Im_, 2N^−^}
Cu,ZnSOD	0.0018	100		

The charges of species have been omitted to more clarity

## Summary

In this study we presented preliminary results describing the ability of the cyclic peptide c(HKHGPG)_2_ to form Cu(II)/Zn(II) complexes depending on pH. It has been shown that above pH 6 the Cu(II)/Zn(II) heteronuclear complex are formed. For [CuZnL]^4+^, [CuZnH**-**_1_L]^3+^, [CuZnH**-**_2_L]^2+^, [CuZnH**-**_3_L]^+^ species molecular modeling were performed in order to characterize the structural aspects of selected complexes. The main objective was to verify whether the dominant complex at physiological pH may well reproduce the active center of Cu,ZnSOD, whether they are able to inhibit free radical reaction. Therefore, we examined the SOD enzymatic activity for the complexes existing in 7.4 pH for the following systems: Cu(II)/c(HKHGPG)_2_/Zn(II), Cu(II)/c(HKHGPG)_2_, and Cu(II)/c(HKHGPG)_2_/Cu(II). The coordination abilities for equimolar and double excess of Cu(II) ions have been described and characterized in the earlier work (Kotynia et al. 2014). The comparison of the IC_50_ values of c(HKHGPG)_2_ complexes in different systems, shows that the best inhibitory activity of superoxide anion exhibits [CuZnL]^4+^ complex. This complex dominates in at the physiological range of pH, wherein Cu(II) is bound by {2N_Im_, 2N^−^} and Zn(II) ions with the {2N_Im_, 2H_2_O} coordination manner. In systems with only Cu(II) ions (at pH 7.4) prevailing species have the same coordination sphere around Cu(II) ions and the constant stability of this complexes are very similar. Therefore, superior enzymatic activity can be affected by the simultaneous presence of Zn(II) ions in complex.
